# Editorial: Addressing Roles for Glycans in Immunology Using Chemical Biology

**DOI:** 10.3389/fchem.2020.00471

**Published:** 2020-06-11

**Authors:** Matthew S. Macauley, Christoph Rademacher, Karina V. Mariño

**Affiliations:** ^1^Departments of Chemistry, Medical Microbiology and Immunology, University of Alberta, Edmonton, AB, Canada; ^2^Department of Biomolecular Systems, Max Planck Institute of Colloids and Interfaces, Potsdam, Germany; ^3^Laboratorio de Glicómica Funcional y Molecular, Instituto de Biología y Medicina Experimental (IBYME-CONICET), Buenos Aires, Argentina

**Keywords:** glycans, carbohydrates, glycoimmunology, glycan binding protein (GBP), chemical biology

## Introduction to Chemical Glycoimmunology

Advances in genetic techniques have enabled the identification of disease-susceptibility genes and the development of experimental models for mechanistic studies. However, this progress has only partially translated into new therapies, highlighting the relevance of both epigenetic control of gene expression and post-translational modifications as control points in the transition from genotype to phenotype. The cell surface provides a tightly-regulated temporal and spatial signature containing crucial biological information, and much of its dynamic response to environmental factors remains to be explored.

Glycoconjugates, macromolecules containing carbohydrates (glycans) linked to proteins or lipids, are a central component of the cell surface. These structurally diverse biomolecules expose their carbohydrate portion outside the cell, and in doing so, are essential in storing and transmitting information and regulating cell-cell interactions. One of the many fields where glycans have arisen as key communicators is immunology, as they mediate diverse immunological functions (Zhou et al., 2018). The complex mechanisms involved in the biosynthesis and assembly of glycans is a non-template driven manner, which results in a high, natural heterogeneity that is key to adapting cellular responses. However, structural characterization of these glycans and the functional interpretation of the array of carbohydrates on a cell surface, generally referred to as the glycome, remains an analytical challenge for chemists.

Since the glycome is unique to each cell type and significantly different between organisms, “self” and “non-self” signals are imbedded in the glycome (Varki, 2011). Moreover, glycans on immune cells are involved in different processes such as cell differentiation, trafficking, and response to pathogens (Varki, 2017). In this sense, glycan information is translated by glycan binding proteins (GBPs), also called lectins. The abilities of these GBPs to modulate immune cell function is intimately connected to their ability to differentiate glycan structures in a precise and effective manner. Evolution of lectins has granted these proteins a selective recognition, which is influenced not only by structure, but also by multivalency of their cognate glycan ligand, reflecting the naturally crowded and heterogeneous state of the cell surface (Kaltner et al., 2019). Consequently, chemistry has been crucial in untangling the roles of GBP-glycan interactions. Chemical tools and approaches provide a powerful complementary approach that has greatly aided our understanding of GBPs by designing inhibitors and synthetic ligands able to control immune cell function via glycan recognition. Below, we discuss the 13 manuscripts within this series in the context of three themes: GBPs, immunomodulation, and technological advances.

## Targeting and Exploiting GBPs

In mammals, immune cells are modulated by three major GBP families: C-type lectins (Brown et al., 2018), Galectins (Cerliani et al., 2017), and Siglecs (Duan and Paulson, 2020; Laubli and Varki, 2020). Galectins are a family of evolutionary-conserved, soluble lectins involved in multiple immunoregulatory pathways. Unraveling the molecular determinants of ligand recognition has and will continue to provide a path toward lectin engineering for improved function and efficient inhibition. This topic is reviewed by Modenutti et al., providing a synopsis of the available tools for studying galectins and their structural characterization, with emphasis on the role of water molecules in galectin-glycan binding. An alternative for modulating immune responses is using recombinant lectins to target cell surface glycans. Working with an engineered galectin1-galectin3 tetramer, Farhadi et al. investigated induction of apoptosis in cultured T cells. Through amplifying and integrating galectin-1 and−3 activities, these synthetic constructs provide new tools for understanding how these lectins, and their glycan ligands, influence innate and adaptive immunity. Beyond the human immune response, Vasta et al. provide a review on simpler organisms lacking adaptive immunity that also leverage galectins. Focusing on oysters and a parasite called *Perkinsus marinus*, that can deplete oyster stocks, the authors review recent evidence implicating galectin-like proteins produced by the oysters as a defense mechanism against the parasite. An intriguing mechanism is proposed whereby these oyster-derived GBPs bind both “self” and “non-self” glycans on host and pathogen to bring the parasite to the phagocytic hemocytes of the host.

Even though lectin families are defined by their affinity toward certain monosaccharides or oligosaccharide structures, most GBPs have low monovalent affinities, which is greatly enhanced through cell surface multivalency. Inspired by this principle of avidity, Li et al. and Goyard et al. designed synthetic multivalent glycoclusters to target GBPs on mammalian immune cells or pathogens, respectively. In the first manuscript, the focus is on DC-SIGN, a C-type lectin on dendritic cells (Li et al.). A comprehensive 20-member library of mannosides was synthesized based on 5 substructures of a high mannose N-glycan, each displayed at four valencies, and the most multivalent (hexameric) constructs showed the best binding to DC-SIGN. A TLR agonist and peptide T cell epitope were conjugated to the hexameric constructs and, surprisingly, their ability to induce monocyte maturation and T cell activation did not directly correlate with ligand potency. Accordingly, the avidity for DC-SIGN may have other consequences, such as intracellular routing and antigen presentation. In the second manuscript, a synthetic strategy is presented to prepare heteroglycoclusters designed to interact with LecA (galactose-binding lectin) and LecB (fucose binding lectin) (Goyard et al.). These lectins are virulence factors from *Pseudomonas aeruginosa*. Working with cyclopeptide-based heteroglycoclusters, fucose, mannose, and galactose can be co-presented to mimic the natural cell surface diversity and simultaneously target multiple lectins.

## Immunomodulation Using Glycoconjugates or Manipulating Glycans

Two major classes of glycoproteins are N-glycans and mucin-type O-glycans (Moremen et al., 2012). Heavily O-glycosylated mucins are an important component of the molecular shield of gastrointestinal, respiratory, and reproductive epithelial tissues. Pinzón Martín et al. reviewed the role of mucins in immunomodulation from a chemical perspective, pointing out the current challenges in their synthesis, expression, and structural characterization as fundamental and necessary steps toward understanding their clinical application. Glycoproteins can also be used for antibody development of anti-carbohydrate antibodies, which often requires the glycan to be chemically conjugated to a carrier protein. Pillot et al. address an important question, asking whether the protein conjugation site matters. Working with the Pneumococcal surface adhesin A protein, five mutants were engineered each with a single cysteine at different distances from known T-helper epitopes. A synthetic tetrasaccharide carbohydrate antigen, from the capsular polysaccharide of *S. pneumoniae*, was conjugated to the mutants via maleimide chemistry. The conjugation site furthest from the T-helper epitopes generated the highest anti-carbohydrate antibody titers, indicating that the site of conjugation does matter, which has important implications for the design of conjugate vaccines.

Glycolipids are another major class of glycoconjugates. Work by Lai et al. and Howlader et al. demonstrate applications of synthetically-prepared glycolipids and inhibitors of glycolipid-acting enzymes, respectively. Invariant NKT (iNKT) cells recognize glycolipids presented by CD1d (Rossjohn et al., 2012), and Lai et al. explore how iNKT cells respond to α-Lactosyl Ceramide (α-LacCer) relative to the commonly used α-Galactosyl Ceramide (α-GalCer). α-LacCer was synthesized and found to stimulate much weaker iNKT cell responses compared to α-GalCer. Consequently, α-LacCer suppressed iNKT responses to α-GalCer, which the authors use as a means of modulate iNKT responses and disease in the context of several iNKT-dependent injury mouse models. In the second manuscript, the ability of catabolic enzymes (sialidases) to remodel glycolipids were examined in the context of leukocyte adhesion. Using a neuraminidase-3 (Neu3) inhibitor, Howlader et al. report that interactions between the integrin LFA1 and ICAM, a key step in leukocyte adhesion and migration to inflammatory sites, is altered. Modulating Neu3 activity not only affected cell glycolipid composition, but also altered LFA1 sialylation, expression on the cell surface, and lateral mobility.

## Technological Advances for Studying Immune Cell-Related Glycoconjugates

Chemistry has been instrumental in generating libraries of natural oligosaccharides. Such libraries have enabled the study of GBP specificity and glycoprofiling of diverse, complex biological samples, such as human plasma to investigate aberrant glycosylation as a source of disease biomarkers. Specifically, Glycan libraries continue to be strongly leveraged for use in glycan microarrays. A review of glycan microarrays is presented by Gao et al., which begins by exploring chemical linking strategies used for immobilizing glycans to solid supports. An overview is presented on applications of glycan microarrays for studying the glycan binding specificity of Siglecs and Galectins, entry receptors for pathogens, and naturally occurring anti-glycan antibodies in humans (Gao et al.). Next generation approaches, such as those using DNA-encoding and cell-based approaches, are also discussed.

The natural heterogeneity of glycans has, for many years, delayed their detailed structural characterization and impaired a thorough understanding of their biological relevance. Three articles deal with the analytical challenge of glycan characterization from different perspectives (Lippold et al.; Rebello et al.; de Haas et al.). de Haas et al. review the advances in understanding the role of N-glycans on cell surface immune receptors. Here, available methodologies are discussed in relation to their potential to study this post-translational modification on processes such as pathogen recognition, antigen presentation, and immune signaling. Considering that monoclonal antibodies as therapeutics have revolutionized clinical approaches to treat many diseases, and the fact that their glycosylation can regulate their therapeutic efficacy through modulating their interactions with antibody receptors, glycoprofiling of antibodies has reached the spotlight in recent years (Mimura et al., 2018). Lippold et al. present the development of an affinity chromatography-mass spectrometry approach to understand the relevance of heterogeneous glycosylation of monoclonal antibodies. Their methodology focuses in the heterogeneous glycosylation of mAbs and its interaction with FCgIIIA, which is critically important in antibody-dependent cellular cytotoxicity. This functional separation was applied to Cetuximab as a case study, since this antibody contains a glycosylation site in both the Fc and Fab regions. Finally, and beyond biotherapeutics, the human plasma N-glycome has arisen as a source of biomarkers for different pathologies. Rebello et al. present a mass spectrometry assay for the relative quantitation of antennary fucosylation in total plasma N-glycome. The utility of this application was highlighted by its ability to detect and quantify antennary fucosylation in the plasma of colorectal cancer patients.

## Future Perspectives

This collection of articles highlights the relevance of chemical approaches for studying glyco-related immune pathways, exemplified with probes to target GBPs, structure–function relationships, and new analytical techniques. A close collaboration and interplay between chemists and immunologists have and will continue to lead to new breakthrough in the roles of glycosylation in immunity. Indeed, outcomes of this interdisciplinary strategy is facilitating and will certainly accelerate vaccine design, identification of new biomarkers and moreover, precise modulation of biotherapeutic activities.

## Author Contributions

All authors listed have made a substantial, direct and intellectual contribution to the work, and approved it for publication.

## Conflict of Interest

The authors declare that the research was conducted in the absence of any commercial or financial relationships that could be construed as a potential conflict of interest.
